# Combined thrombogenic effects of vessel injury, pregnancy and procoagulant immune globulin administration in mice

**DOI:** 10.1186/s12959-020-00245-8

**Published:** 2020-11-07

**Authors:** Yanqun Xu, Yideng Liang, Leonid Parunov, Daryl Despres, Michael Eckhaus, Dorothy Scott, Mikhail Ovanesov, Evi B. Struble

**Affiliations:** 1 Center for Biologics Evaluation and Research, U.S. Food and Drug Administration, 10903 New Hampshire Ave, Silver Spring, MD 20993-0002 USA; 2 Mouse Imaging Facility, National Institutes of Health, Bethesda, MD USA; 3 Pathology Service, Division of Veterinary Resources, National Institutes of Health, Bethesda, MD USA

**Keywords:** Factor XIa, FXIa, Thrombogenicity, Immune globulin, Thrombosis

## Abstract

**Background:**

Pregnant women are at increased risk of thrombotic adverse events. Plasma derived immune globulin (IG) products, which are used in pregnancy for various indications, may contain procoagulant impurity activated coagulation factor XI (FXIa). Procoagulant IG products have been associated with increased thrombogenicity but their effect in pregnancy is unknown.

**Methods:**

Late pregnant (gestation days 17–20) or early lactation (days 1–3) and control female mice were treated with IGs supplemented with human FXIa then subjected to ferric chloride (FeCl_3_) vessel injury. Occlusion of blood vessel was assessed by recording blood velocity in the femoral vein for 20 min using doppler ultrasound laser imaging. FXIa dose was selected by the ability to increase thrombin generation in mouse plasma in vitro.

**Results:**

FXIa produced robust thrombin generation in mouse plasma ex vivo. Following FeCl_3_ injury, pregnant and non-pregnant mice receiving IG + FXIa exhibited faster reduction of blood velocity in femoral vein compared to IG alone or untreated controls. In vitro, thrombin generation in plasma samples collected after thrombosis in FXIa-treated animals was elevated and could be reduced by anti-FXI antibody.

**Conclusions:**

Our results suggest that intravenously-administered FXIa may contribute to thrombosis at the site of vascular injury in both pregnant and non-pregnant animals.

**Supplementary Information:**

**Supplementary information** accompanies this paper at 10.1186/s12959-020-00245-8.

## Introduction

Normal pregnancy is a hypercoagulable condition that mitigates the risk of bleeding during delivery. These changes to the hemostatic system also put pregnant women at risk of thrombotic events (TEs) including deep venous thrombosis (DVT), pulmonary embolism, cerebral vein thrombosis [1, 2], myocardial infarction and stroke [3, 4]. The incidence of DVT in pregnancy is 5 to 12 per 10,000, and pregnancy confers a 7 to 10-fold increased DVT risk compared to age-matched non-pregnant women [5, 6]. In the U.S., thrombotic pulmonary embolism accounts for 10% of pregnancy-related deaths [7]. Long-lasting morbidities include post-phlebitis syndrome after DVT (20–50%) and chronic thromboembolic pulmonary hypertension after PE (5%) [8–10].

Immune globulin (IG) products are used in moderate to high doses in pregnancy to prevent recurrent pregnancy loss, complications of anti-phospholipid syndrome, neonatal alloimmune thrombocytopenia, post-partum relapses in relapsing-remitting multiple sclerosis, and as treatment for primary immunodeficiency and various autoimmune diseases. Hyperimmune IG products have also been proposed for prevention of vertical transmission of cytomegalovirus and Hepatitis B viral infection [11, 12].

In 2010, US and European regulatory authorities in collaboration with industry identified that elevated activated Factor XI (FXIa) levels in a specific IG product were associated with increased risk of thrombosis [13–16]. Procoagulant effects of TE-implicated batches in vitro and in vivo were abrogated by selective depletion of FXIa, prompting changes to manufacturing processes that resulted in IG products with an improved safety profile [13]. The thrombogenic role of FXIa has long been suspected in other clinical situations [17, 18]. Early generations of plasma-derived FXI concentrates caused severe TEs in FXI deficient patients [19], whereas those with reduced levels of FXIa did not activate coagulation in human studies [17]. FXI activation has been demonstrated in blood from patients with aortic stenosis, chronic obstructive pulmonary disease and ischemic cardiomyopathy [20–23].

Association of pregnancy with thrombosis following IG therapy has not been reported to date and studies in gravid animals have not been performed. Although most in vivo animal research, including coagulation and hemostasis studies, are performed in mice, there is little to no information about whether pregnant mice (or any other species) recapitulate the hypercoagulable state of human pregnancy [24]. Compared with in vitro studies, an in vivo model should capture the dynamics and three-dimensional extent of clot formation in the setting of physical effects of blood flow, and dynamic replenishment of pro- and anti-coagulant factors. In this work, we studied pregnant and control mice to compare the thrombogenic risks of infusions of non-procoagulant IG with or without added FXIa. We did not find significant differences in FeCl_3_ -induced femoral vessel occlusion between pregnant and non-pregnant mice, suggesting that in this species, pregnancy does not result in higher propensity for FXIa mediated prothrombotic propensity. However, treatment with FXIa resulted in faster blood velocity reduction in vivo and higher thrombin generation in vitro and ex vivo. We unexpectedly found that FXIa activity persists in circulation, despite presence of proteins known to inactivate it.

## Materials and methods

### Materials

Human plasma-derived FXIa (for some in vitro studies), corn trypsin inhibitor (CTI), inhibitory monoclonal anti-FXI(a) antibody (clone anti-FXI-2, catalog# AHXI-5061) and enzyme inhibitor PPACK were from Haematologic Technologies Inc. (Essex Junction, VT). A glycerol-free preparation of human FXIa for in vivo experiments was from Enzyme Research Laboratories (South Bend, IN). Recombinant lipidated tissue factor (TF) was either Recombiplastin® from Instrumentation Laboratory pt?>(Bedford, MA) or Innovin® from Dade Behring (Marburg, Germany). Tissue Factor (TF) activity was determined using the Actichrome TF chromogenic kit (American Diagnostica, Lexington, MA). Fluorogenic substrate for thrombin Z-Gly-Gly-Arg-AMC was from Bachem (Torrance, CA). Phospholipid vesicles were from Technoclone (Diapharma, West Chester, OH). The Thrombinoscope® Thrombin Calibrator was from Stago (Parsippany, NJ).

#### Commercial IG products

IG drugs were purchased from the NIH Pharmacy in Bethesda, MD during 2012, 2013 and 2014 and used within their labeled shelf life periods.

#### Commercially available animal and human plasma

Pooled normal mouse and human plasma were purchased from Lampire Biological Laboratories (Pipersville, PA) and Innovative Research Inc. (Novi, MI). In some experiments, frozen human normal pooled, affinity-depleted FXI deficient (Affinity Biologicals Inc., Ancaster, ON, Canada) or congenital FXI deficient (George King Biomedical Inc.) plasma were used.

#### Evaluation of FXIa activity in procoagulant IG products in vitro

In-house Thrombin Generation Assay (TGA) was performed to characterize IG products as described in Liang et al [25] with minor modifications. Specifically, human FXI-deficient plasma (50% vol/vol) was mixed with fluorogenic substrate for thrombin (800 μM), phospholipid vesicles (4 μM), TF (0.3 pM), and serially diluted IG samples or calibrator FXIa. In-house software was used to calculate parameters of thrombin generation response after recalcification with calcium chloride (12.5 mM). Maximal rate of fluorescent increase (also known as thrombin peak height) was used as assay readout.

#### Evaluation of FXIa-induced thrombin generation in animal plasma in vitro and ex vivo

A 96 pipettor-based micro-volume variant of the TGA for evaluation of animal plasma samples was based on method of Shibeko et al. [26]. Briefly, animal plasma (30 μL, 50% vol/vol) were set up in wells of a conical well microplate followed by addition of 24 μL of a mixture of CTI (100 μg/mL), phospholipid vesicles (4 μM), TF (0.5 pM), and either FXIa, anti-FXI(a) antibody (50 μg/mL), or buffer. The experiment was initiated using a 96-channel micropipettor (Hydra DT, Thermo Fisher Scientific, Hudson, NH, USA) which dispensed 50 μL of plasma mixture into all 96 wells of another conical microplate preloaded with 5 μL of fluorogenic substrate for thrombin (800 μM), and CaCl_2_ (12.5 mM), followed up by the transfer of 40 μL of activated reaction mixture into a half-area 96-well plate for reading inside a temperature (37 °C)-controlled microplate reader Biotek Synergy H4 (BioTek Instruments, Inc., Winooski, VT, USA). To estimate the dependence of TG on the presence of FXIa, plasma samples were tested twice on the same assay plate, with and without added anti-FXI(a) antibody. For ex vivo experiments, thrombin activity was additionally calibrated with Thrombinoscope® Thrombin Calibrator using in house version of CAT software as described in Chang et al. [27]. To assess method variance, three pools of normal mouse plasma were tested in 32 wells each, producing CVs of 5, 6 and 17%. The described method was sensitive to detect increased TG in human pooled plasma mixed with at least 2 pM of human FXIa.

#### Evaluation of FXIa thrombogenicity in mice

The animal protocol and procedures within were approved by the FDA Institutional Animal Care and Use Committee (AUCUC) and all methods were performed in accordance with the relevant guidelines and regulations. To obtain timed-pregnant animals, female SJL-E mice (Charles River Laboratories, MA) were placed into a male’s cage overnight and, the next day, checked for the presence of vaginal plugs. If a plug was found, the females were presumed pregnant, separated from the male and the designated as pregnant on gestation day (GD) 1.

To assess general tolerability of the selected non-procoagulant and procoagulant IG preparation in pregnant mice, a pilot study was performed. For this, two pregnant (GD18 ± 2) or non-pregnant mice per group received intravenous injections of non-procoagulant IG (npIG, Hizentra® CSL Behring, King of Prussia, PA, nominal IG concentration 200 mg/mL) alone or spiked with 0.5, 5 or 50 IU FXIa/mL (equivalent of 0.3, 3 or 30 pmol/mL) (npIG+ FXIa). The IG dose administered was 1 g/kg (5 mL/kg or 150 uL/30 g mouse); the associated FXIa dose was 1.5, 15, and 150 pmol/kg. Two pregnant and non-pregnant mice received negative control (saline) at the same dose-volume as the IG injection. For this, mice were pre-warmed for up to 2 min using a heat lamp to promote aid vasodilation and then placed in mouse restrainers. One of the lateral caudal veins was identified and sterilized with chlorhexidine scrub and alcohol swab. Test articles were injected using a 27G 5/8″ needle attached to a tuberculin syringe. Blood was collected from the submandibular vein immediately following test article administration and via cardiac puncture at termination. Animals were observed for acute toxicity two times on the day of injection (immediately following the administration and up to 6 h after), and twice daily during the first week after administration. The litters were observed at birth and then at the end of study for viability and general health. Gross pathology of all the animals that received test article and histopathology of the dams was performed at the end of the study, 1 week following test article administration.

#### Imaging of FeCl_3_-induced thrombosis in mice

The pilot study was followed by in vivo Doppler imaging experiments in the presence of vessel injury. The animal protocol and the procedures within were approved by FDA IACUC, all methods were performed in accordance with the relevant guidelines and regulations, and the personnel completed training required by FDA/CBER and NIH. Female SJL-E mice (Charles River Laboratories) aged 10–25 weeks on late gestation (GD17–20) or early lactation (lactation day (LD) 1–3), or age matched non-pregnant controls were weighed and placed under general anesthesia in an induction chamber with 4–5% isoflurane delivered by gas mixed with oxygen, medical air and nitrogen. Once the mice were unconscious, they were transferred to a nose-cone, maintained with 1–2% isoflurane and kept warm by external heated pads and by the warmer on the physiological monitoring board which records ECG, respiratory rate, and temperature. Ophthalmic ointment was applied to the corneas to prevent desiccation while under anesthesia. The animals were then transferred to the physiological monitoring board of the Doppler instrument, femoral vein blood flow on the left hind limb was identified using a High Frequency Doppler (HFD) 32 mHz ultrasound probe under the color mode of the instrument and defined as Area of Interest (AOI). Blood velocity was measured in the pulse wave mode of the instrument at four randomly selected, but consistent positions in the AOI. This served as the baseline blood velocity (v0) for each of the experimental mice. Four groups, each consisting of 8 to 9 mice then received either npIG (1 g/kg) or npIG + FXIa (1 g/kg + 150 pmol/kg) via tail injection as before. An additional 5 to 6 mice received no treatment. Thus, the study groups were (a) non-pregnant npIG controls, *n* = 9; (b) non-pregnant npIG + FXIa, *n* = 8, (c) non-pregnant untreated controls, *n* = 5; (d) pregnant npIG controls, *n =* 8; (e) pregnant npIG + FXIa, *n =* 8; and (f) pregnant untreated controls, *n* = 6. Subsequently, while animals were under deep anesthesia, the femoral vessel was surgically exposed, tissues separated by gentle blunt dissection with a sterile hemostat to expose the femoral vein and, 7–9 min after test treatment or control treatment injection, the vein was covered with filter paper saturated with 3% FeCl_3,_ causing injury to the vessel wall. The filter paper remained in situ for 3 min at which point it was removed and the blood flow at the AOI was recorded as before, every minute until 20 min after filter paper removal.

#### Parameters of blood flow and statistical analysis

The velocity was recorded at a baseline (v0, measured at t0, before injection) and every minute until 20 min after filter paper removal. For each time point, the blood flow velocity values from the four positions in the AOI were averaged. The average velocity data was transformed to percent change from v0; 25 and 50% blood flow reduction were arbitrarily chosen as benchmark levels. Time points corresponding to the benchmark levels (t (25) and t (50) respectively) were estimated for each animal using the approach illustrated in Fig. S1. Briefly, the transformed velocity datapoints bracketing the benchmark value (i.e. immediately above and below 25 and 50% reduction, respectively) were fitted with a linear function (OriginPro 2018 software, OriginLab Corporation). The x times (x) when velocity change (y) equaled − 25 and − 50% were then computed, having been defined as t (25) and t (50), respectively.

To estimate the rate of flow change (acceleration or deceleration of blood flow), the average blood velocity data were first smoothed by adjacent averaging with a window of 3 datapoints (OriginPro 2018) to reduce the effects arising from random fluctuations in recorded data. First derivatives were then computed (OriginPro 2018) and the global minima of the first derivatives (fastest deceleration) were compared for different groups of animals.

For statistical analyses, the animals were grouped by pregnancy state (pregnant and non-pregnant, row factor) and by treatment modality (untreated controls, npIG, and npIG + FXIa, column factor) and the variations in t (25), t (50) were analyzed by two-way ANOVA (OriginPro 2018). If row factor did not significantly contribute to the variation, then the groups were combined and the differences between treatment groups were analyzed with t-test; Bonferroni correction was introduced for multiple comparisons. For all statistical analyses, *p* < 0.05 was considered significant.

#### Histopathological analysis

At the end of data collection, mice were sacrificed and the femoral vessel at the site of damage was carefully dissected. The tissues thus collected were fixed in 4% formaldehyde for at least 24 h, and then embedded in paraffin and sectioned longitudinally every 75-μm step for a total of 9 steps per specimen. Two sections per each step were collected; one section was stained with hematoxylin and eosin stain and one was stored unstained for future processing. Care was taken to preserve the orientation of the tissue when sections were placed on the slide so that the proximal and distal orientation of the tissues could be easily identifiable. Slides were examined and, for a random subset of animals in each dose group that displayed at least a 50% reduction in blood velocity, i.e. 5 npIG+FXIa and 5 untreated (*n* = 1) or npIG treated (*n* = 4) controls, representative slides were sent to study pathologist for blinded final histopathological analysis.

## Results

### Human FXIa is procoagulant in mouse plasma in vitro

**Fig. 1:**
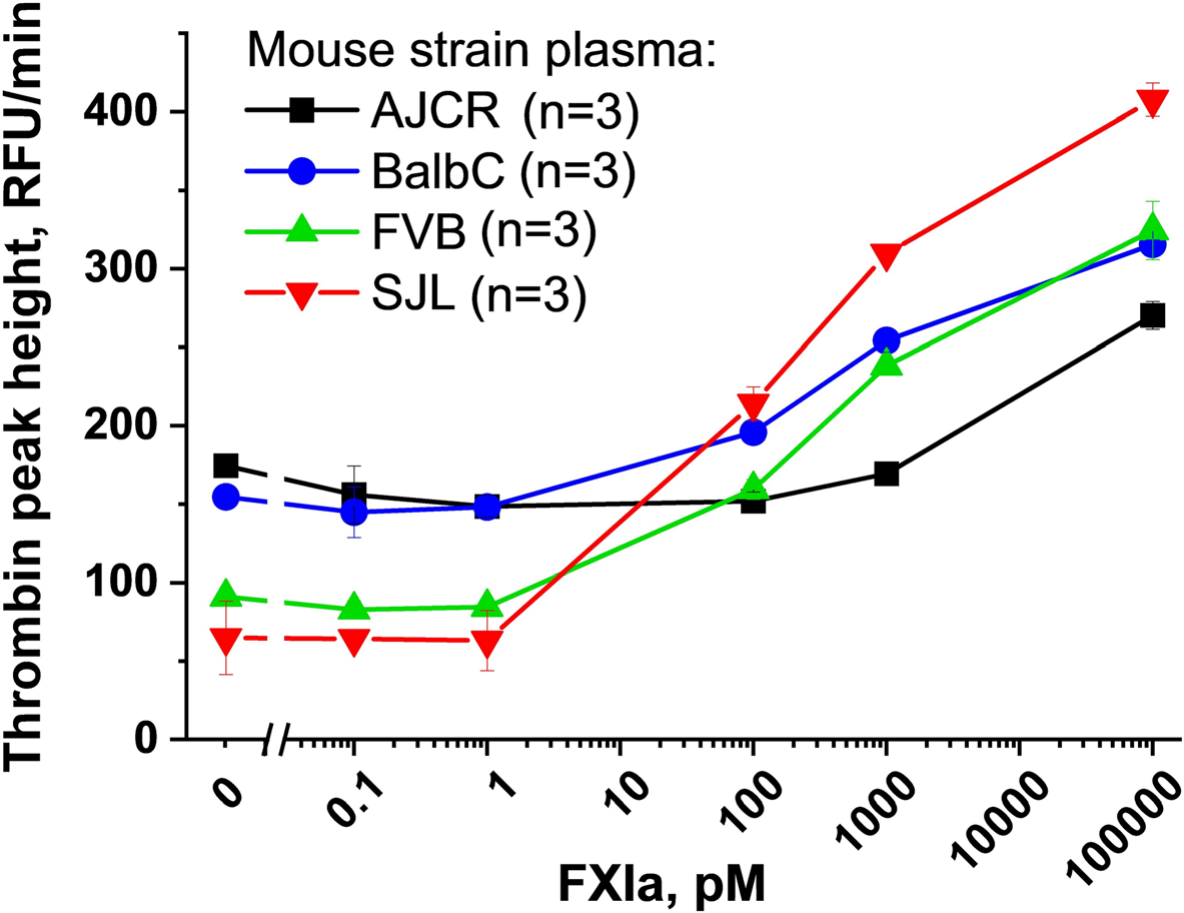
Human FXIa induces thrombin generation responses in mouse plasma. Thrombin formation (y-axis) in plasma of four mouse strains in response to increasing concentrations of human FXIa (x-axis). To ensure good quality of plasma, all samples were collected in-house under identical conditions. SJL strain was selected for the relatively wide range of dose-response in this assay. *n* = 3

To select the strain of mice sensitive to human FXIa, mouse plasma samples were compared in vitro by their ability to produce thrombin generation in response to increasing concentrations of FXIa (Fig. 1). The SJL strain was selected due to relatively wide dose-response range and their large size. Based on the response, for in vivo experiments, non-procoagulant IG spiked with 0.3–30 nM of FXIa was chosen as a model of procoagulant IG product. An un-spiked IG product was chosen as a control treatment.

### Evaluation of procoagulant IG toxicity in the absence of injury

In pilot experiments, 1 g/kg IV injections of immune globulin product spiked with human FXIa at three levels (0.3, 3, and 30 nM, or, respectively, 0.5, 5 and 50 IU FXIa/mL), was tolerated well and without sings of systemic or local toxicity. Non-pregnant and pregnant mice on GD17–20 received the test article in attendance of study pathologist who observed the animals for signs of acute toxicity. Mice were further observed periodically for up to 6 h following the administration and then twice daily till the end of the experiment. No change in posture, gait, breathing, or other outward signs of thrombosis were seen at any point. All mice maintained good body condition until the experimental endpoint, 1 week after the administration. The pregnant mice delivered healthy pups. Necropsy of the dams was performed 1 week after injection and tissues were collected for histopathological analysis. Gross and microscopic examinations by a certified pathologist did not reveal signs of thrombosis, except possible weak signal at the highest FXIa doses. Similarly, administration of negative control treatments, npIG and saline, was well tolerated and not associated with toxicity.

Overall, the IV administration of neither procoagulant IG, nor negative control treatments resulted in signs or symptoms of thrombosis.

### Blood flow imaging after FeCl_3_ injury

Further experiments were conducted on animals subjected to local, FeCl_3_-induced, blood vessel injury, resulting in thrombosis initiation. For this, the blood flow from the femoral vein in left hind limb (Supplemental Fig. S2) was visualized by live imaging using a HFD ultrasound probe under the color mode of the instrument and defined as Area of Interest (Supplemental Fig. S3). The accuracy of the identification of AOI during the experiment was ensured by preliminary measurements of stable blood flow before inducing the injury, and clear separation between signals deriving from the vein and artery, respectively. Furthermore, the selected location was easily accessible to induce local venous injury with minimal collateral tissue damage.

**Fig. 2:**
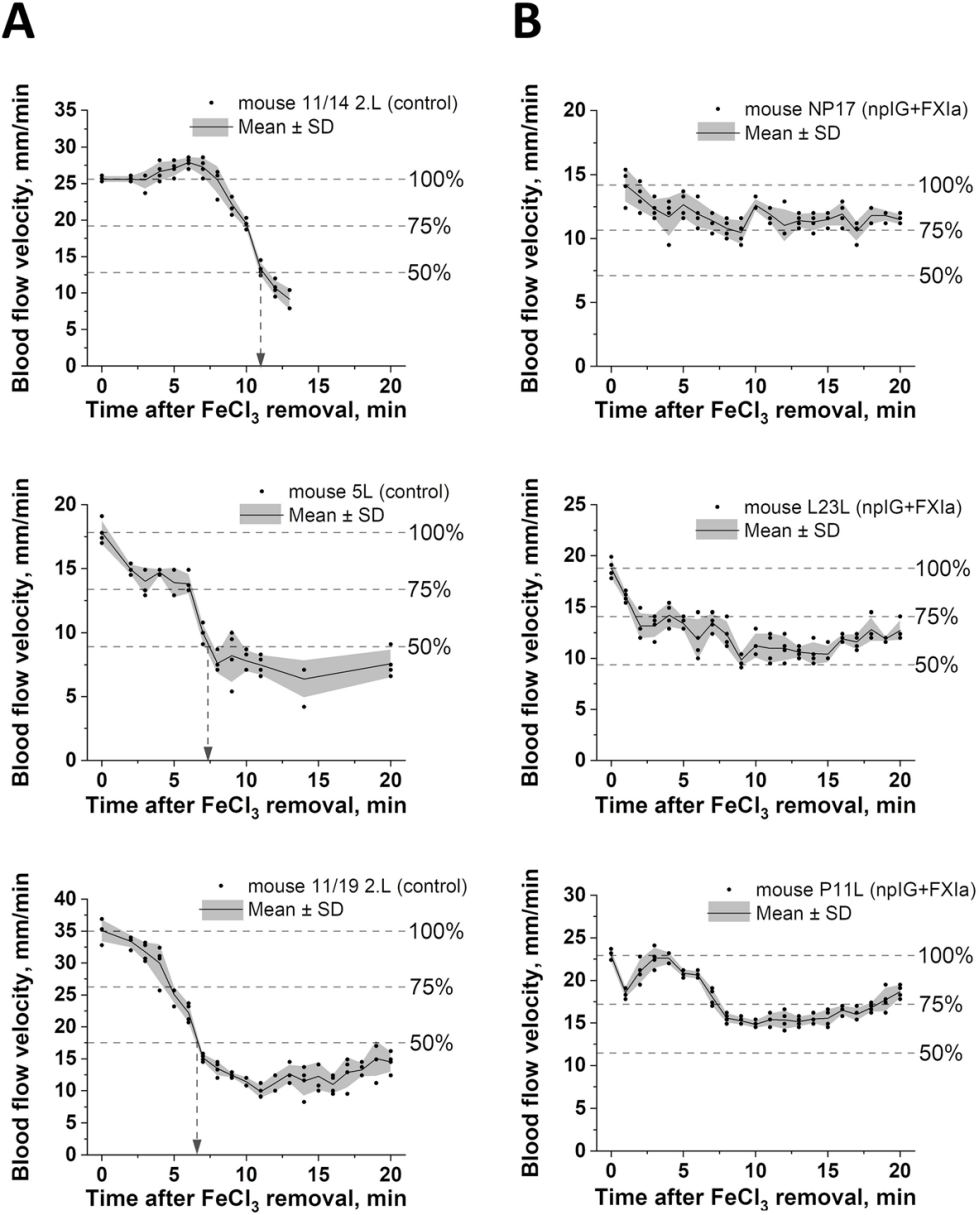
Examples of blood flow kinetics in individual animals measured with a Doppler ultrasound probe after FeCl_3_ induced femoral vein injury in mice. Shown are data from *n =* 3 mice that did (**a**) or did not (**b**) reach 50% reduction in blood flow velocity within 20 min

Two types of responses to FeCl_3_ injury were observed in our studies. In most experiments, blood flow velocity declined sharply within 20 min of FeCl_3_ (Fig. 2a). In other experiments blood flow velocity started to decline and then reached a plateau or even bounced back, possibly indicating slower flow obstruction or embolization of the thrombus or its parts (Fig. 2b).

**Fig. 3:**
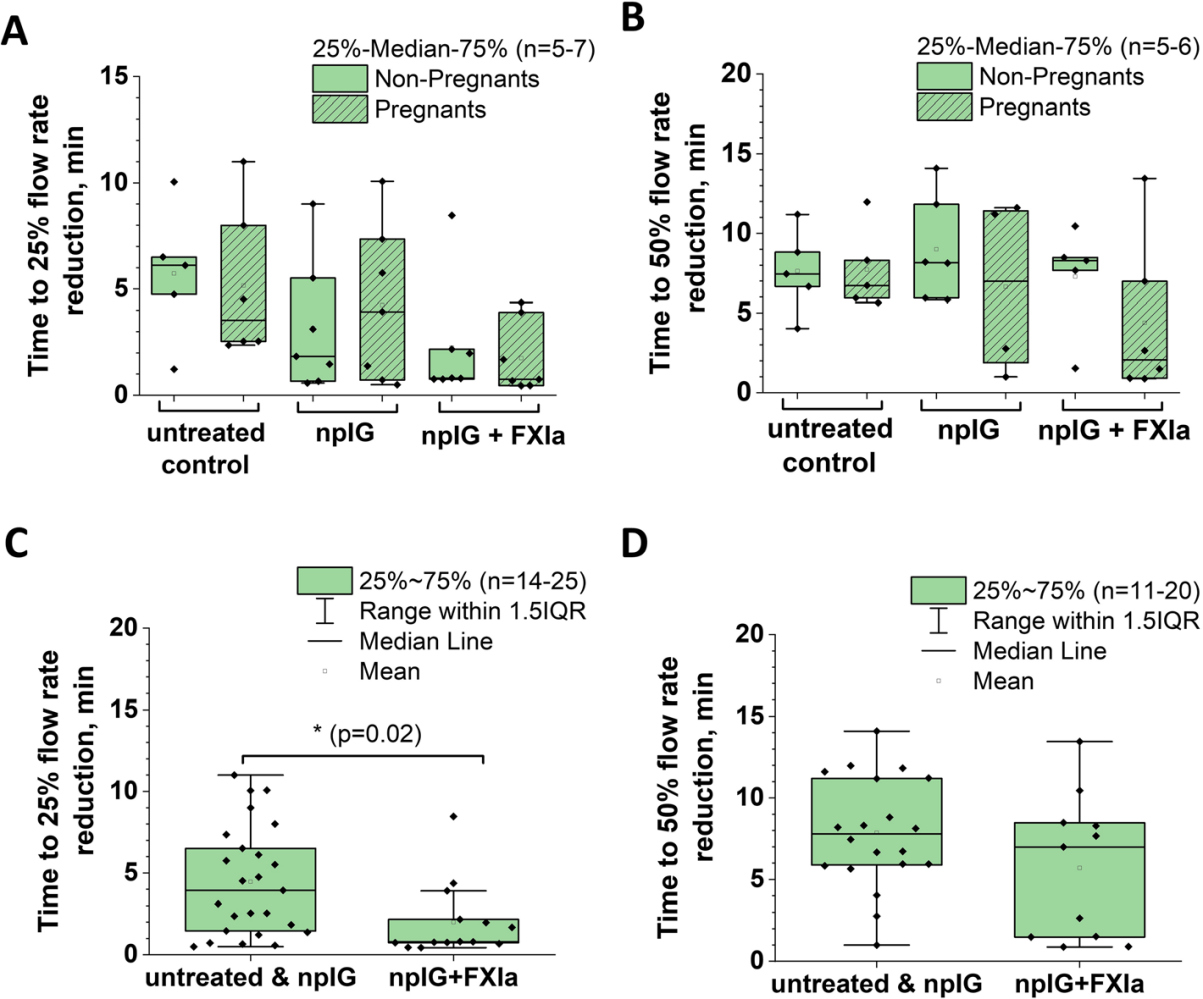
Time to 25 and 50% blood flow reduction in femoral vein after FeCl_3_ induced injury. **a** and **b** Blood flow reduction in mice stratified by treatment and pregnancy status. *n* = 5–7. **c** and **d** Blood flow reduction in the mice stratified by treatment only *n* = 11–25

To assess the thrombogenic state in all experimental animals, including those that exhibited blood flow recovery by 20 min, we defined and analyzed two parameters, t (25) and t (50), i.e. the time to achieve 25 and 50% of blood flow reduction, respectively. Both parameters displayed a trend towards a reduction after administration of FXIa-containing IG material (Fig. 3a and b). Two-way ANOVA analysis of these parameters grouped by pregnancy status (row factor) and treatment status (no treatment, npIG and npIG+FXIa, column factor) revealed that pregnancy does not significantly contribute to the variation (*p* = 0.96 and 0.21, for t (25) and t (50), respectively). When data were analyzed irrespective of the pregnancy status, the group receiving npIG did not differ from untreated animals (not shown, *p* = 0.2 and 0.8 for t (25) and t (50) respectively). When untreated and npIG animals were pooled together, t (25) was significantly lower in group receiving FXIa compared to control and npIG animals (*p* = 0.02, t-test) (Fig. 3c); t (50) differences, although trending lower in FXIa treated animals, did not reach significance when analyzed using t-test (*p* = 0.13, Fig. 3d).

**Fig. 4:**
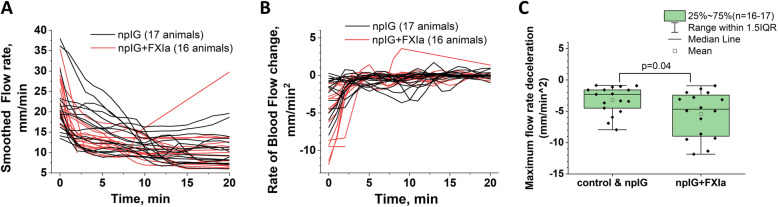
Blood flow velocity and the rate of blood flow change in animals treated with npIG or npIG+FXIa irrespective of their pregnancy status. **a** Blood flow velocity kinetics for all mice (*n* = 16–17) after applying a smoothing function (see methods for details)). **b** Rate of blood flow change; data represents first derivative of flow data presented on panel A (*n =* 16–17). **c** Minimum of first derivative (maximum of blood deceleration) observed within 20 min of experiment (*p* = 0.04, *n =* 16–17)

We also analyzed the highest rate of blood flow change among animals treated with npIG and npIG+FXI. For each animal data for blood flow velocity were smoothed, differentiated and plotted to visualize the rate of blood flow change (Fig. 4a and b). The global minimum of blood flow change was significantly lower in the group receiving FXIa (*p* = 0.04) (Fig. 4c) indicating the faster rate of blood flow reduction.

### Histopathological analysis of FeCl_3_ injured blood vessels

**Fig. 5:**
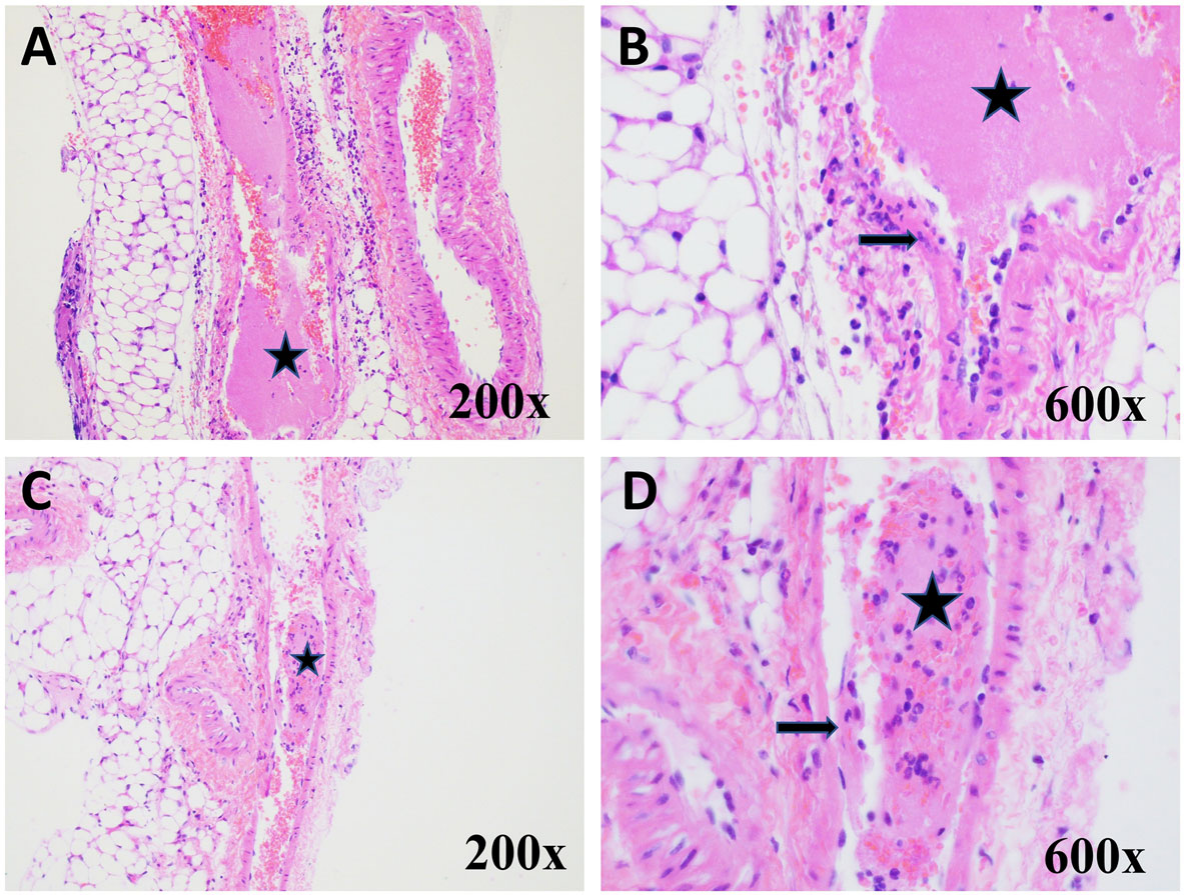
Images of thrombi observed in the femoral veins of mice following FeCl3 injury. **a**. Control mouse -Longitudinal section of femoral artery and vein. Lumen of vein is occluded by a large fibrin thrombus (asterisk) admixed with red blood cells. Hematoxylin and Eosin (H&E) 200x. **b**. Control mouse - Higher magnification of distal aspect of femoral vein demonstrating damage to vessel wall (arrow) with loss of endothelial and smooth muscle cells and focal infiltrate of neutrophils within wall. H&E 600x. **c**. FXIa treated mouse - Longitudinal section of femoral artery and vein demonstrating partially occlusive thrombus (asterisk). H&E 200x. **d**. FXIa treated mouse - Higher magnification of femoral vein thrombus (asterisk) comprised of fibrin, red blood cells and neutrophils. Damage to the adjacent wall of vessel (arrow) with loss of endothelial and smooth muscle cells. H&E 600x

At the end of blood flow data collection, mice were sacrificed, and the femoral vessel at the site of damage was carefully dissected, stained with H&E and examined microscopically (Fig. 5). Representative slides from five animals reaching at least 50% reduction in blood velocity from each group (npIG or sham control and npIG+FXIa) were sent for blinded histopathological analysis. The analysis confirmed the presence of either a large thrombus or a small thrombus in most of the veins observed (9/10 vein fragments). Specifically, left femoral veins from the control group animals were found to have a small (2/5 mice), medium (1/5 mice) or large (2/5 mice) thrombus. The presence of the thrombus was accompanied with damaged endothelium (4/5), characterized by missing endothelial cells, damaged endothelial wall, and loss of smooth muscle along a portion of vein adjacent to thrombus. 4/4 animals that displayed endothelial damage (with or without the presence of a large thrombus) had also displayed velocity reduction by 50% or more, and maintained it for 20 min, whereas one mouse without noted endothelial damage (but with one small thrombus present) did not.

Similarly, 2/5 np + FXIa treated mice had a large and 2/5 a small thrombus. However, one mouse from this group showed no thrombus, but instead the vein was dilated, and blood filled. No endothelial damage was evident in this animal or in the 2/5 animals from this group that had small thrombi. All 5/5 mice from this subset maintained 50% reduction in blood velocity at 20 min. This suggests that prominent endothelial damage is not the only mechanism for thrombus formation in this group. Neutrophil infiltrates were also observed close to the vessels in 3/5 animals. The possibility exists that an early thrombus, once present in this location, may have been dislodged from the location. Although unlikely, given that the femoral triangle of vein, artery and nerve is clearly identifiable in all the slides, human error in tissue collection and processing cannot be ruled out.

### Procoagulant activity detected in mouse plasma ex vivo after FXIa administration

**Fig. 6:**
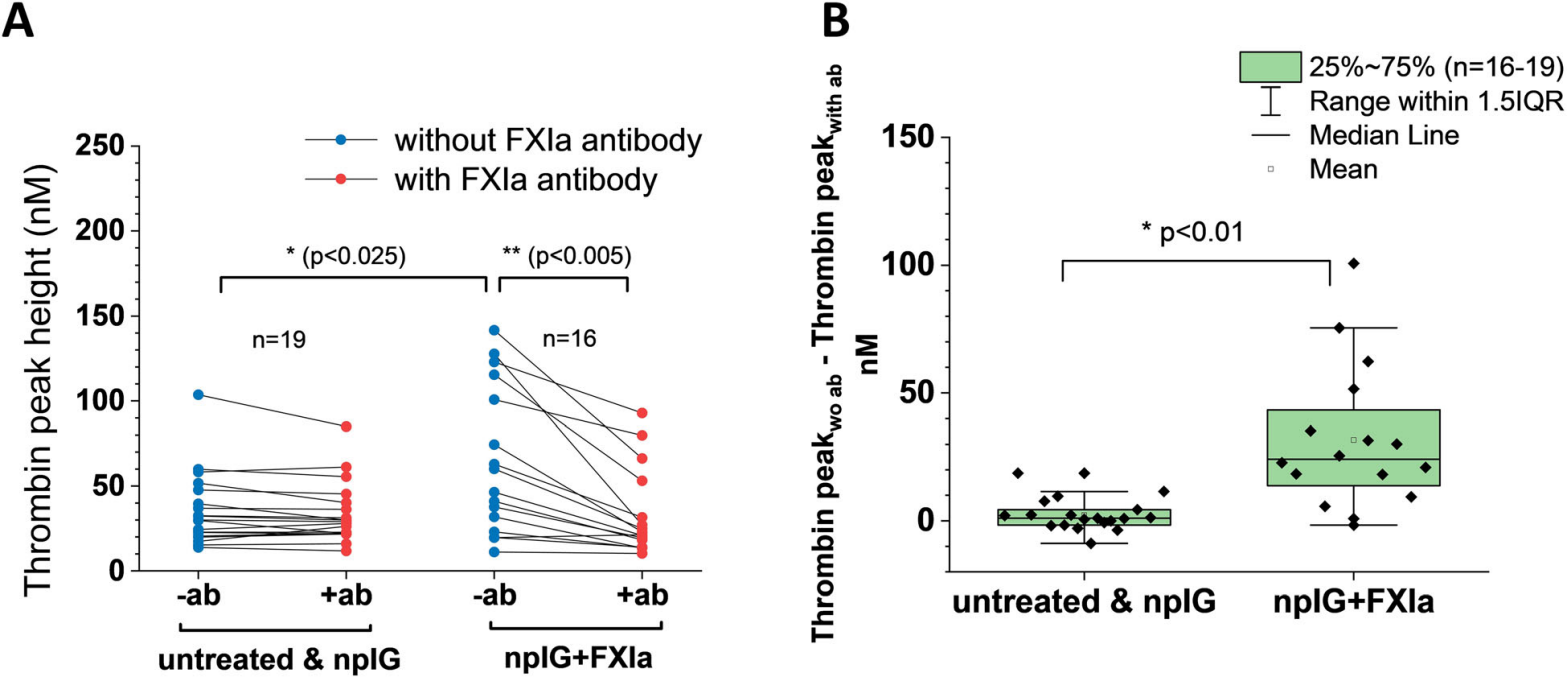
Higher thrombin generation response in vitro in plasma collected 1–2 h after FXIa administration. **a** Thrombin peak height in terminal mouse plasma (collected at the conclusion of the FeCl_3_ experiments), paired experiments performed with and without antibody for FXIa. In the absence of the antibody, animals that received npIG+FXIa have significantly higher thrombin generation in vitro compared to those that did not receive FXIa (untreated and treated with npIG). The addition of the antibody for FXIa significantly reduced thrombin generation in the former, but not in the latter, suggesting FXIa-dependent procoagulant activity that persists for the duration of the study. **b** Absolute difference in thrombin peak height in terminal mouse plasma following the addition of anti-FXIa antibody. For each animal the difference in respective thrombin peaks before and after adding anti-FXIa antibody is calculated.*, significant difference, unpaired 2 sample t-test, Bonferroni correction for multiple comparisons; **, significant difference, paired t-test

To confirm presence of FXIa in animals at the end of FeCl_3_ experiment, blood samples were collected and tested in TGA under two conditions: with and without anti-FXIa antibody (Fig. 6a).

In the absence of antibody, samples from animals treated with npIG+FXIa produced significantly more thrombin compared to the control group (untreated and treated with npIG) (Fig. 6a). This procoagulant potential was significantly inhibited in the presence of antibody for FXIa (Fig. 6a, *p* < 0.005, paired t-test).

The absolute difference in thrombin peak height for paired experiments, i.e. the difference in thrombin without and with antibody, was significantly higher for the npIG+FXIa group compared to the control group (Fig. 6b). These results suggest that FXIa can remain in circulation for the duration of experiment.

## Discussion

Thromboembolism is a common cause of pregnancy-related maternal death in the U.S. [7]. Deep venous thrombosis may also cause long-term morbidity from post-phlebitis syndrome [8–10]. IG products with procoagulant activity may increase the risk of thrombotic events in the hypercoagulable state of pregnancy, but the risk has not been assessed. Thus, there is a need for human or animal studies to characterize the safety of IG products in this important population.

Previous studies identified FXIa contamination as a biochemical root cause for IG-related TEs and suggested that manufacturing modifications in purification processes as well as appropriate thrombogenicity testing may improve safety of IG products. The goal of our investigation was twofold, a) to assess if pregnant mice could be used to model the hypercoagulable state of human pregnancy and b) to characterize the toxicity of FXIa contaminated IG administration. Although we did not observe increased coagulation propensity in pregnant mice under the conditions of our experiments, our findings in all study animals taken in toto help explain the observed clinical picture in humans.

Similar to humans, no toxicity of FXIa impurity in healthy animals was seen. This observation is consistent with the relatively low rates of thrombotic events in humans. Most TE-implicated batches of IG were associated with one to three TE reports despite being administered to hundreds or thousands of patients each. This suggests contribution of other procoagulant risk factors in some patients, e.g., vascular lesions or other procoagulant conditions [28].

Several factors may explain the lack of significant FXIa toxicity in mice. First, interspecies differences in the blood flow and hormonal status could result in thrombogenic differences between humans and mice both in pregnancy and after FXIa treatment. For example, some studies have demonstrated relevance of mouse pregnancy, pregnancy failure, as well as changes in coagulation system to humans [29, 30], but while others have highlighted differences, such as in activated Protein C resistance during pregnancy [31]. In our in vitro thrombin generation experiments we also observed differences between human and mouse plasma. Notably mouse plasma is less sensitive to human FXIa than plasma collected from humans. Based on these data we used higher doses of human FXIa in mice compared to the amounts found in TE-implicated batches. However, even under these species-adjusted levels, FXIa administration did not result in thrombosis, with or without pregnancy state. A second factor that may explain the lack FXIa toxicity in this study is the administration of FXIa spiked IG products rather than known thrombogenic IG product batches. Products associated with TEs during 2010 and 2011 were removed from market, thus they were not available for this study.

At present the evidence to implicate vascular injury in thrombosis after IG administration is limited. We hypothesized that vascular dynamic parameters following FeCl_3_-induced vessel injury will be different after administration of npIG+FXIa compared to npIG or no treatment. We expected to find occlusive thrombi after every application of the FeCl_3_ – saturated filter paper over the femoral vessels, and that was what we observed in some of the animals. However, a fraction of the animals exhibited slow thrombus growth, incomplete occlusion and even re-canalization of the femoral vein within 20 min. One potential reason for these variations (other than individual animal differences) could be the relatively low concentration of FeCl_3_ (3%) used to induce vessel damage. Under the conditions of our experiments, higher concentrations of FeCl_3_ resulted in very rapid thrombosis and even mortality. Whatever the reason, the FeCl_3_ model did not meet our expectations with regards to the expected outcome.

Despite this, the analysis of the real-time blood velocity data collected pointed to a difference between control animals and those receiving FXIa. In presence of vascular injury, administering FXIa increased the propensity for thrombus formation and rapid growth as indicated by: (1) faster decline in blood velocity in the injured vein (assessed by t (25), *p* = 0.02 and (2) faster deceleration of blood flow (*p* = 0.04).

Although the precise mechanism of thrombus formation following FeCl3 injury in mice is not known, it is believed that free radicals and damage to endothelial cells is involved, culminating in thrombogenesis. One investigation implicated several components of the coagulation system in this model including basal membrane exposure, changes in adhesive proteins such as fibrinogen and von Willebrand factor, and promotion of platelet adhesion [32]. Wang et al demonstrated that FeCl_3_ venous and arterial thrombogenesis is FXI dependent with FXI deficient mice displaying lower rates of thrombosis [33–35].

Finally, we found that FXIa activity could be detected 2 h after intravenous administration in mice, suggesting slow removal of this procoagulant impurity from circulation. This finding could explain the observation that venous and arterial thrombotic events often occurred hours after completion of IG infusion.

Our findings provide important justification for heightened control of procoagulant activity in products administered in pregnancy. The procoagulant state in pregnancy is multifactorial and incompletely understood (reviewed in [1, 2]). Altered levels of pro- and anticoagulant factors, venous stasis, vascular injury, and thrombophilia may provide multiplicative rather than additive contributions to overall thrombotic risk [36, 37].

## Supplementary Information

**Additional file 1.**

## Data Availability

The datasets used and/or analysed during the current study are available from the corresponding author on reasonable request.

## Abbreviations

AOI: Area of Interest
AUCUC: Animal Care and Use Committee
CTI: Corn trypsin inhibitor
ECG: Electrocardiogram
FDA: U.S. Food and Drug Administration
FXIa: Coagulation factor XIa
GD: Gestation day
HFD: High Frequency Doppler
IG: Immune globulin
IU: International unit
IV: Intravenous
TF: Tissue factor
TGA: Thrombin generation assay
