# Supplementary material for: Combined thrombogenic effects of vessel injury, pregnancy and procoagulant immune globulin administration in mice
Source: Thromb J. 2020 Nov 7;18:32. doi: 10.1186/s12959-020-00245-8 (PMC7648396; doi:10.1186/s12959-020-00245-8)
Supplement: Supplementary file 1 — Additional file 1. [file 12959_2020_245_MOESM1_ESM.docx]

Supplementary materials

**Title:** Combined thrombogenic effects of vessel injury, pregnancy and procoagulant immune globulin administration in mice

Yanqun Xu^1^, Yideng Liang^1^, Leonid Parunov^1^, Daryl Despres^2^, Michael Eckhaus^3^, Dorothy Scott^1^, Mikhail Ovanesov^1,#^**,** Evi B. Struble^1,&^

**Participating Researchers:**

**Author Affiliations:**

^1^Center for Biologics Evaluation and Research, U.S. Food and Drug Administration, Silver Spring, MD;

^2^Mouse Imaging Facility, National Institutes of Health, Bethesda, MD;

^3^Pathology Service, Division of Veterinary Resources, National Institutes of Health, Bethesda, MD

**Contact information:**

**^#^** [mikhail.ovanesov@fda.hhs.gov](mailto:mikhail.ovanesov@fda.hhs.gov) (corresponding author); Tel (office): (240) 402-7301,

^&^[evi.struble@fda.hhs.gov](mailto:evi.struble@fda.hhs.gov), (240) 402-7403

10903 New Hampshire Ave, WO 52/72-4206, Silver Spring, MD 20993-0002


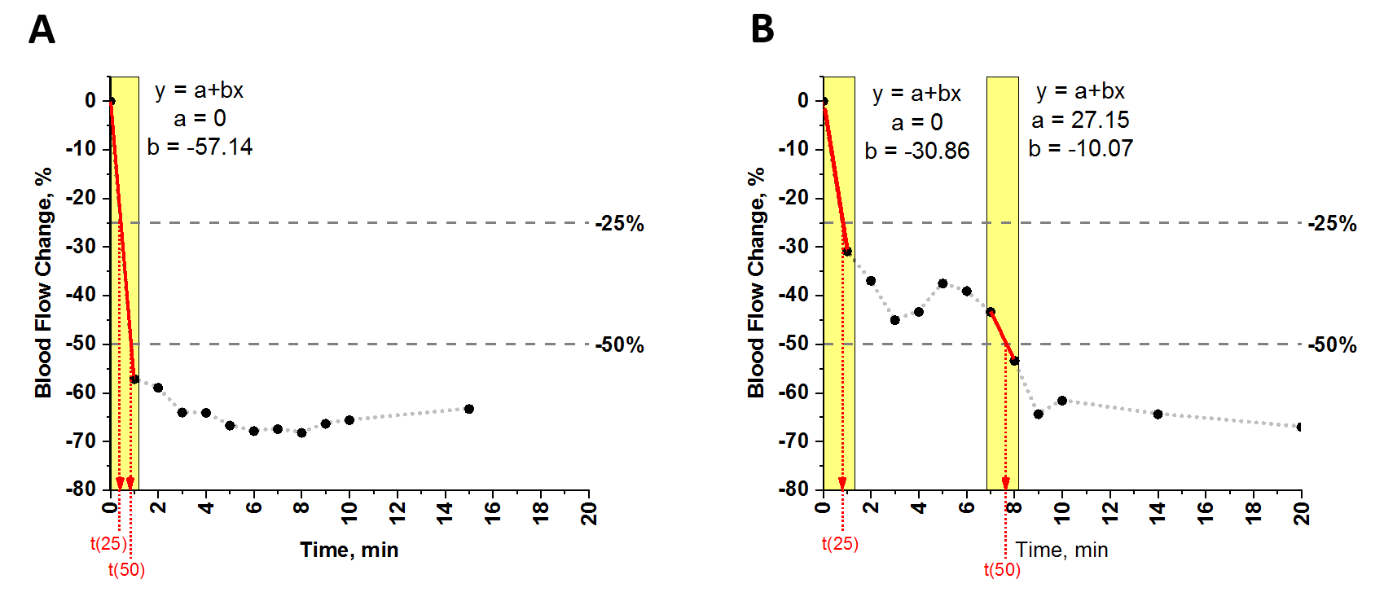


Supplemental Figure S1. Computing time to 25 and 50% blood flow reduction (t(25) and t(50) respectively). Two benchmark levels were arbitrarily chosen to correspond to 25 and 50% blood flow reduction (broken line at -25 and -50 in the y axis) from baseline. To find t(25) and t(50), the datapoints bracketing the benchmark values were fitted with a linear function (red line). Two possible scenarios are shown, (A) a single line equation (same bracketing datapoints) for both benchmark values, and (B) two different line equations, one for each benchmark value. The time (x) when the line(s) intersected y=-25 and y=-50 were then computed and represented t(25) and t(50), respectively.


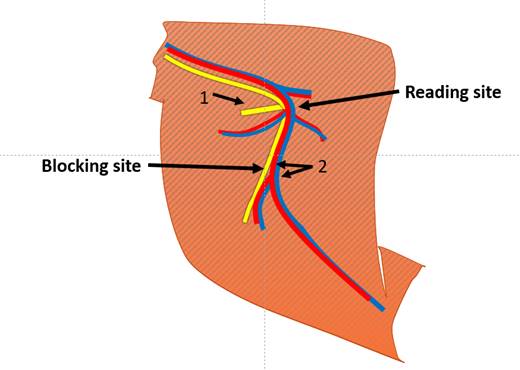


Supplemental Figure S2. Schematic drawing of the site of injury (Blocking Site) and area where the blood flow was recorded (Reading Site). Red color represents arteries, blue veins, and yellow nerve. Marked are femoral nerve (1) and femoral artery and vein (2).


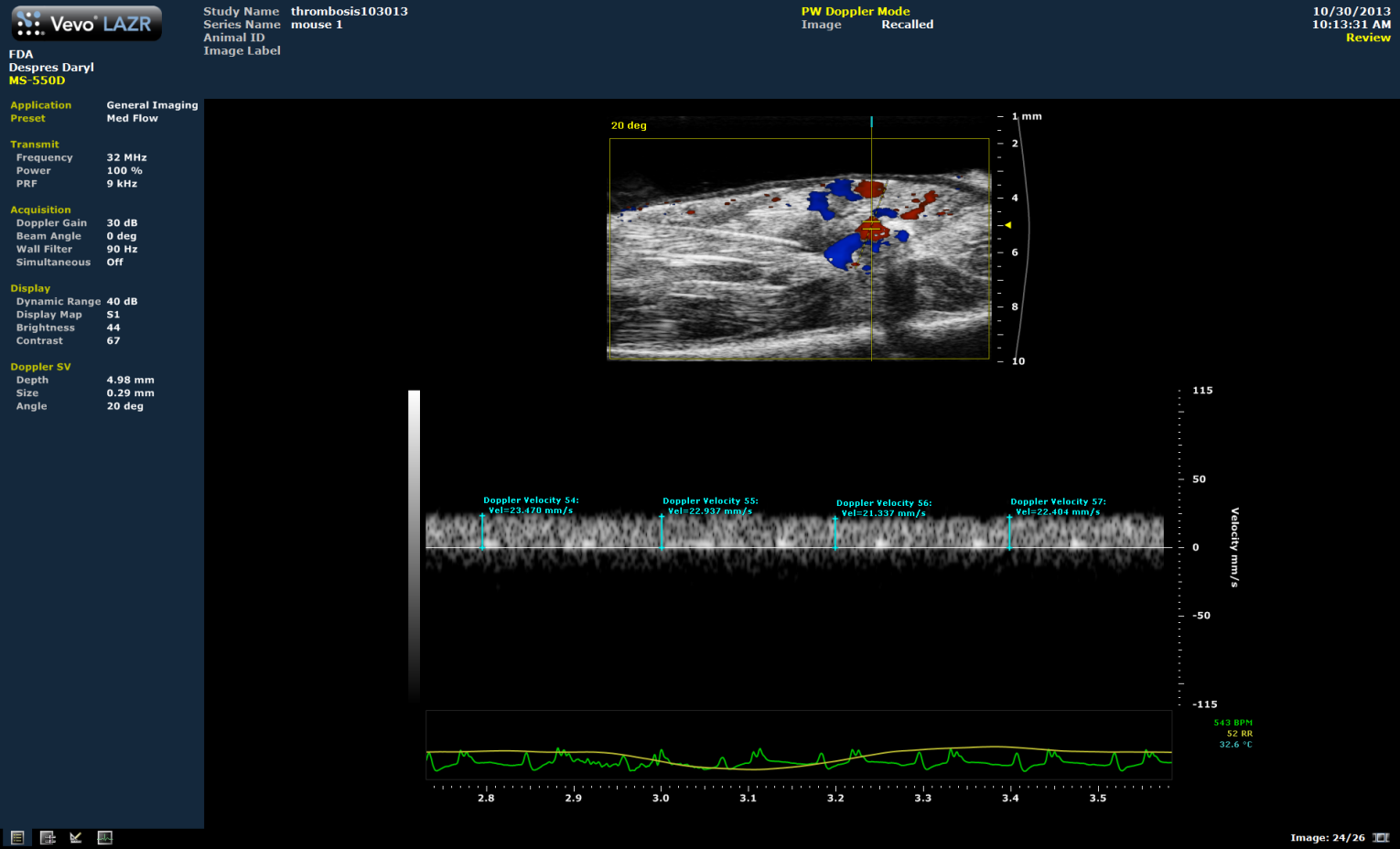


Supplemental Figure S3. Femoral blood flow with Doppler Ultrasound. Snapshot of Doppler data collection under PW mode at the NIH Mouse Imaging Center. Vertical cursor indicated the Area of Interest where the velocity was measured.
